# Securing an OTL-HNS residency: how competitive is it? Comparing medical student perceptions to actual Canadian statistics

**DOI:** 10.1186/s40463-017-0192-4

**Published:** 2017-02-27

**Authors:** E. Kay-Rivest, N. Varma, G. M. Scott, J. J. Manoukian, M. Desrosiers, J. P. Vaccani, L. H. P. Nguyen

**Affiliations:** 1 Department of Otolaryngology – Head and Neck Surgery, McGill University, Montreal, Canada; 2 Department of Otolaryngology – Head and Neck Surgery, Western University, London, Canada; 3 Department of Otolaryngology – Head and Neck Surgery, Université de Montréal, Montréal, Canada; 4 Department of Otolaryngology – Head and Neck Surgery, University of Ottawa, Ottawa, Canada; 5 Center for Medical Education, McGill University, Montreal, Canada

**Keywords:** Competitiveness, Residency match, Medical education

## Abstract

**Background:**

The residency match is an important event in an aspiring physician’s career. Otolaryngology – Head and Neck Surgery (OTL-HNS) is a surgical specialty that has enjoyed high numbers of applicants to its residency programs. However, recent trends in Canada show a decline in first-choice applicants to several surgical fields. Factors thought to influence a medical student’s choice include role models, career opportunities and work-life balance. The notion of perceived competitiveness is a factor that has not yet been explored. This study sought to compare competitiveness of OTL-HNS, as perceived by Canadian medical students to residency match statistics published yearly by CaRMS (Canadian Residency Matching Service), with the hope of informing future decisions of surgical residency programs.

**Methods:**

An electronic survey was created and distributed to all medical students enrolled in the 17 Canadian medical schools. After gathering demographic information, students were asked to rank what they perceived to be the five most competitive disciplines offered by CaRMS. They were also asked to rank surgical specialties from most to least competitive. Publically available data from CaRMS was then collected and analyzed to determine actual competitiveness of admissions to Canadian OTL-HNS residency programs.

**Results:**

1194 students, from first to fourth year of medical school, completed the survey. CaRMS statistics over the period from 2008 to 2014 demonstrated that the five most competitive specialties were Plastic Surgery, Dermatology, Ophthalmology, Emergency Medicine and OTL-HNS. Among surgical disciplines, OTL-HNS was third most competitive, where on average 72% of students match to their first-choice discipline. When students were questioned, 35% ranked OTL-HNS amongst the top five most competitive. On the other hand 72%, 74% and 80% recognized Opthalmology, Dermatology and Plastic Surgery as being among the five most competitive, respectively. We found that fourth-year medical students were significantly more knowledgeable about the competitiveness of both OTL-HNS and Plastic Surgery compared to first-year students (*p < 0.01*).

**Conclusion:**

Overall, Canadian medical students may underestimate the competitiveness of OTL-HNS. Furthermore, competitiveness would appear to be a concept that resonates with medical students during the match process.

## Background

The residency match is widely considered one of the most important events in an aspiring physician’s career. Many factors have been shown to influence a medical student’s choice of residency program, including presence of positive role models, long-term career opportunities, perceived work-life balance, heaviness of call schedule, academic and research opportunities, and length of post-graduate training [1–12]. To varying degrees, these considerations can either attract or deter students from applying to certain residency programs.

Otolaryngology – Head and Neck Surgery (OTL-HNS) is a surgical specialty that, historically, has enjoyed high numbers of applicants to its residency programs. However, first-choice applicants to this discipline in Canada have declined recently, mirroring a decline experienced in other surgical disciplines. National data from the Canadian Residency Matching Service (CaRMS), for the period of 2002-2007 compared to 2008-2013, showed a 16.1% decline in first-choice applicants to OTL-HNS, similar to declines seen in neurosurgery (23.1%), urology (18.0%) and plastic surgery (15.1%) [13, 14]. Despite this decline, overall OTL-HNS remains one of the most competitive disciplines offered.

A recent commentary published by Kaplan et al. described the possibility that competitiveness in our specialty may affect applications to OTL-HNS programs. Indeed, they noted that 80% of Boston Medical School students described matching to OTL-HNS as either “impossible” or “near impossible” [15]. With this in mind, we decided to interrogate Canadian medical students, in order to assess their perceptions of OTL-HNS competitiveness.

To our knowledge, the notion of perceived competitiveness of a specialty as a potential influencing factor has not yet been explored in the medical education literature. This study sought to determine if Canadian medical students over- or under- estimate the competitiveness of OTL-HNS residency programs as compared to the match statistics published yearly by CaRMS, with the hope that it may inform future decisions of surgical residency programs.

## Methods

### Ethics, consent and permissions

McGill University’s institutional review board granted ethical approval for this study. In addition, written consent was obtained from CaRMS to quote their online statistics.

### Perceived competitiveness

An electronic cross-sectional survey was created by the research team, with the aim of exploring Canadian medical students’ perceptions of competitiveness in applying to OTL-HNS residency programs. The survey was created through the Questionpro software, was accessible both in English and French, and was entitled “*Competitiveness of Canadian Residency Programs*”. The title was left intentionally broad in order not to highlight the OTL-HNS focus and to diminish any potential bias. Participation in the survey was purely voluntary, all responses were kept strictly anonymous, and participants could withdraw from the study at any time. Students were required to fill an online informed consent form prior to gaining access to the survey.

The e-survey was distributed via Facebook to all medical students (year one to four) enrolled in any of 17 Canadian medical schools. Contact was made with each medical school, which then posted the survey link onto its class’ common Facebook page. The schools contacted can be found in Appendix 1. The survey was published on November 10^th^, 2014 and was closed on February 25^th^, 2015.

The survey captured basic demographic information from participants (i.e. age, gender, level of training). Students were asked to rank what they perceived to be the five most competitive disciplines offered by CaRMS (chosen from an alphabetically ordered list of all specialties available). Finally, students were asked to consider only surgical specialties, and to rank them from most to least competitive. The designation of a specialty as “surgical” was predetermined as posted on the CaRMS website. The exception was Obstetrics and Gynecology, which was included as a surgical specialty for the purposes of our study (due to the large technical skills component) despite not being considered a surgical subspecialty by CaRMS.

### Actual competitiveness

Publically available data from the CaRMS website was collected and analyzed to determine actual competitiveness of admissions to Canadian OTL-HNS residency programs in comparison to other surgical and non-surgical specialties. The following data points were collected for the period between 2008 and 2014: total number of applicants; applicants to that discipline only; number of students for whom the discipline was the first choice; number of students matching to their first choice discipline; quota per program, and unfilled positions per program [14]. Competitiveness was defined as the percentage of first-choice applicants gaining entry into their discipline of choice.

### Data analysis

Descriptive statistics were used to analyze the quantitative portion of the survey. The difference in accuracy of the competitiveness of OTL-HNS was examined using a Chi-square test. A *p*-value of 0.05 or less was considered statistically significance. The data was computed using Medcalc version 12.2.

## Results

The survey was begun by 1788 students, and fully completed by 1194 students. Demographic information is summarized in Table 1. There are approximately 11, 500 medical students in Canada, indicating an estimated response rate of 10.4%.

**Table 1:** Currently enrolled year of medical school, gender, highest level of prior education of survey respondents

Year of medical school	
First	13.1%
Second	26.0%
Third	19.1%
Fourth	41.8%
Gender	
Male	40.6%
Female	58.5%
Education prior to medical school	
Pre-med	15.6%
Undergraduate degree	63.8%
Master’s degree	14.4%
Doctoral degree	2.3%
Other	3.8%
Projected Career Choice	
Family Medicine	20.3%
Internal Medicine	11.6%
Surgerical Discipline	22.3%
Otolaryngology (*n* = 40)	3.0%
Not sure	11.6%

### Actual competitiveness

**Fig. 1:**
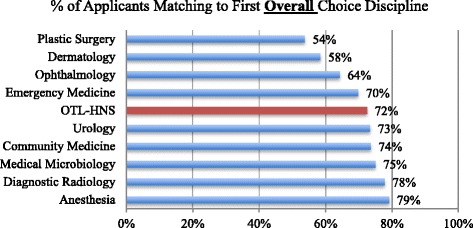
All Disciplines: Average Percentage of Medical Students Matching to Their First Choice Discipline (2008-2014)

**Fig. 2:**
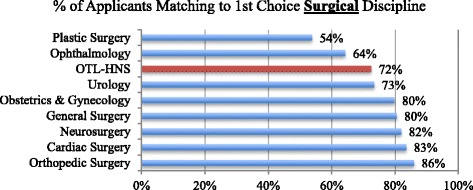
Surgical Disciplines: Average Percentage of Medical Students Matching to Their First Choice Surgical Discipline (2008-2014)

CaRMS statistics over the period from 2008 to 2014 show that the five most competitive specialties were Plastic Surgery, Dermatology, Ophthalmology, Emergency Medicine and OTL-HNS (Fig. 1) [14]. Furthermore, when we examined the ten surgical disciplines, we found that OTL-HNS was third most competitive, where on average, only 72% of students match to their first-choice discipline [14]. Urology had a very similar match rate to OTL-HNS, and Plastic Surgery and Ophthalmology consistently had lower match rates (Fig. 2) [14].

### Perceived competitiveness

Our first question to students after gathering demographic information was: “In your opinion, what are the top five most competitive residency disciplines? Please rank in order (1^st^ most competitive, 2^nd^ most competitive etc.)” Students then had access to a list of the 32 residency disciplines are offered by CaRMS yearly through an alphabetically ordered drop-down menu. In total, 35% ranked OTL-HNS amongst the top five most competitive. On the other hand 72%, 74% and 80% recognized Opthalmology, Dermatology and Plastic Surgery as being among the five most competitive, respectively.

The second question we asked was: “Please rank the following ten surgical disciplines from 1 (most competitive) to 10 (least competitive)”. Students then had access to a list of the ten disciplines and had to give a numerical value to each specialty from 1 to 10. Table 2 summarizes the results. Plastic Surgery received the average score closest to 1 (most competitive), and OTL-HNS was perceived as third most competitive with the third highest average score of 4.8.

**Table 2:** Average Ranking of Perceived Most Competitive Surgical Discipline based on a 10 point Likert scale (1 = most competitive, 10 = least competitive)

Surgical Discipline	Average Score
Plastic Surgery	2.4
Ophthalmology	3.2
OTL-HNS	4.8
Neurosurgery	5.0
Vascular Surgery	6.2
Urology	6.3
Cardiac Surgery	6.4
Orthopedic Surgery	6.6
General Surgery	7.0
Obstetrics and Gynecology	7.0

Finally, we compared the accuracy of assessments based on different demographic information. We found that fourth-year medical students were significantly more knowledgeable about the competitiveness of both OTL-HNS and Plastic Surgery compared to first-year students (*p < 0.01*). A complete comparison of the accuracy of first to fourth year medical students in predicting the competitiveness of different specialties can be found in Table 3. Females were also more likely to have an accurate notion of the competitiveness of OTL-HNS (*p <* 0*.*01). We found no significant difference between undergraduate and master’s students (*p = 0.89*). Students interested in OTL-HNS were significantly more aware of its competitiveness compared to students interested in family medicine (*p <* 0.01).

**Table 3:** Accuracy in rating between first and fourth year medical students

Discipline	Year	% of students aware that this discipline was ranked top 5 overall	*P*-value
OTL-HNS	Med 1	18%	<0.001
	Med 4	37%	
Ophthalmology	Med 1	77%	0.082
	Med 4	69%	
Dermatology	Med 1	77%	0.096
	Med 4	70%	
ER	Med 1	33%	0.194
	Med 4	38%	
Plastic surgery	Med 1	48%	<0.001
	Med 4	76%	

## Discussion

In both Canada and the United States, securing a residency spot is not an easy feat. However, recent data demonstrates that overall, number of applicants to many Canadian surgical discipline are declining [13]. Our study attempted to quantify perceptions of the competiveness of OTL-HNS in Canada, with one question in mind: could this be affecting the application rates? To our knowledge, this is the first study in the literature to explore the notion of perceived competitiveness of a specialty.

Overall, only 35% of Canadian medical students identified OTL-HNS as being among the five most competitive specialties overall. We found that knowledge of the competitiveness of surgical specialties was shown to increase with years of study. There was a statistically significant increase in accuracy of predicting the competitiveness of both OTL-HNS and Plastic Surgery from year one to year four of medical school.

Multiple studies have attempted to understand factors that attract and deter students from applying to surgical fields. An early study tried to identify elements influencing medical students’ career choices and found that strong role models and academic opportunities attracted students to surgical disciplines [12]. It also found that lifestyle; time commitment and call schedules were likely to deter students from a career in surgery. Other factors attracting students to surgical disciplines, as identified in the literature, were resident interactions, number of cases observed in the operating room (OR), and a feeling of involvement in the OR [1, 3, 9]. A survey by Sutton et al, found that “interest in the specialty” and “work-life balance” were key factors determining decisions [9]. Despite all these factors, none of the previously mentioned studies raised the subject of perceived competitiveness. Indeed, we found that among students questioned only one in three students was aware that OTL-HNS was ranked among the top five most competitive disciplines overall. This is different than the study by Kaplan et al. in the United States which showed that students were extremely aware of the difficulty of matching to this specialty [15]. We hypothesize that this lack of awareness could be due to a lack of exposure to the specialty. Certain specialties, such as General Surgery, are mandatory for all students at clerkship level. On the other hand, specialties such as OTL-HNS and Urology are usually only offered as electives in most Canadian medical schools. It could be hypothesized that this may explain the lack of awareness of competitiveness of both surgical disciplines.

Until recently, medical students had very little exposure to these specialties outside of selective rotations. Previous research in plastic surgery has posited that insufficient exposure is contributing to the decline in number of applicants [16]. With an increased emphasis on undergraduate medical education curriculum development in OTL-HNS [17], more students may have an opportunity to explore this specialty. This rise in exposure may influence the volume of applicants to OTL-HNS.

Despite the aforementioned benefits, there are a few limitations of this study. Our sample, although large, may not fully represent the entire population of Canadian medical students. Although we had respondents from every school, some schools had fewer respondents. Another limitation of our study was the definition of the word “competitiveness”, which we defined as percentage of applicants matching to their first-choice discipline. The competitiveness of a specialty, however, depends on multiple factors.

The present questionnaire cannot assess whether competitiveness attracts or deters a student to pursue OTL-HNS, but it does highlight the fact that Canadian medical students may underestimate the competitiveness of the specialty. Competitiveness would appear to be a concept that resonates with medical students during the match process. To our knowledge, this is the first study that attempts to quantify perceived competitiveness of residency programs. In light of the recent decline in application rates, this work may help inform surgical educators and program directors to best promote their specialty to undergraduate medical learners.

## Conclusion

Two-thirds of Canadian medical students are unaware that OTL-HNS ranks among the five most competitive specialties overall. However, students were accurate in assessing how competitive OTL-HNS was compared to other surgical disciplines. A lack of exposure in undergraduate medical training may influence the overall awareness of the competitiveness of OTL-HNS. Future research is needed to assess whether perceived competitiveness is contributing to the decline in applications to surgical disciplines.

## Appendix

**Table 4:** Number of respondents from each Canadian medical school

University	Number of respondents
University of Alberta	10
University of British Columbia	74
University of Manitoba	98
Memorial University of Newfoundland	55
Dalhousie University	81
McMaster University	89
Northern Ontario School of Medicine	53
Queen's University	116
Western University	48
University of Ottawa	67
University of Toronto	170
Université Laval	76
McGill University	228
Université de Montréal	82
Université de Sherbrooke	37
University of Saskatchewan	28
University of Calgary	54

## Abbreviations

CaRMS: Canadian Residency Matching Service
OTL-HNS: Otolaryngology -- Head and Neck Surgery
